# Partitioning the Aggregation of Parasites on Hosts into Intrinsic and Extrinsic Components via an Extended Poisson-Gamma Mixture Model

**DOI:** 10.1371/journal.pone.0029215

**Published:** 2011-12-22

**Authors:** Justin M. Calabrese, Jesse L. Brunner, Richard S. Ostfeld

**Affiliations:** 1 Smithsonian Conservation Biology Institute, Front Royal, Virginia, United States of America; 2 Helmholtz Centre for Environmental Research-UFZ, Leipzig, Germany; 3 Cary Institute of Ecosystem Studies, Millbrook, New York, United States of America; 4 School of Biological Sciences, Washington State University, Pullman, Washington, United States of America; University of Minnesota, United States of America

## Abstract

It is well known that parasites are often highly aggregated on their hosts such that relatively few individuals host the large majority of parasites. When the parasites are vectors of infectious disease, a key consequence of this aggregation can be increased disease transmission rates. The cause of this aggregation, however, is much less clear, especially for parasites such as arthropod vectors, which generally spend only a short time on their hosts. Regression-based analyses of ticks on various hosts have focused almost exclusively on identifying the intrinsic host characteristics associated with large burdens, but these efforts have had mixed results; most host traits examined have some small influence, but none are key. An alternative approach, the Poisson-gamma mixture distribution, has often been used to describe aggregated parasite distributions in a range of host/macroparasite systems, but lacks a clear mechanistic basis. Here, we extend this framework by linking it to a general model of parasite accumulation. Then, focusing on blacklegged ticks (*Ixodes scapularis*) on mice (*Peromyscus leucopus*), we fit the extended model to the best currently available larval tick burden datasets via hierarchical Bayesian methods, and use it to explore the relative contributions of intrinsic and extrinsic factors on observed tick burdens. Our results suggest that simple bad luck—inhabiting a home range with high vector density—may play a much larger role in determining parasite burdens than is currently appreciated.

## Introduction

Parasites, from nematodes and trematodes to lice and ticks, are typically highly aggregated on their hosts with relatively few individuals hosting the large majority parasites [1]–[3]. Indeed, parasite burdens among hosts are usually described by a negative binomial distribution (NBD) with its characteristic long right tail representing those few highly infected hosts [1], [3]. While many explanations for macroparasite (e.g., helminthes, cestodes, nematodes) aggregation exist, most involve small differences among host in terms of behavior, innate susceptibility, or acquired immune responses being magnified throughout the infection and/or lifetime of the host [1], [4]–[8]. Life-long infections and parasite replication on or in the host tend to increase aggregation, while density-dependent parasite mortality and parasite-induced host mortality work to reduce aggregation [4]. Most arthropod vectors, however, spend only a short time on their hosts and reproduce elsewhere, so these feedbacks have little time to manifest. While variation in extrinsic factors has historically been discussed as a potential cause of aggregation [1], [9], [10], recent studies have focused mainly on identifying the intrinsic host characteristics (e.g., sex, age, activity rates) presumably associated with large parasite burdens [11]–[14].

Understanding the cause(s) vector aggregation on hosts is important because this aggregation can inflate the potential rate of spread of an infection [15], [16]. One widely-cited example is tick-borne encephalitis (TBE), which is caused by a virus transmitted between *Ixodes ricinus* ticks when they co-feed on hosts such as yellow-necked mice, *Apodemus flavicollis* [17], [18]. Most TBE transmission occurs on the hosts with the greatest tick burdens [11]. If public health interventions could target the most infested 20% of hosts, transmission of TBE to humans could be effectively reduced by 75% [11], but similar interventions targeted at random hosts could be expected to have only negligible impact [15], [16]. Thus identifying those hosts responsible for feeding and infecting the most vectors has become a priority and has clear implications for disease management.

Currently two classes of models are applied to study parasite aggregation, neither of which allows direct quantification of the relative contributions of intrinsic and extrinsic factors. Regression-based approaches assuming negative binomial error structure and treating 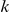, the overdisperison parameter of the NBD, as a nuisance parameter [11], [12], [14] focus on identifying covariates that account for variation in mean burdens among groups of hosts. There is typically no link in these studies between biological processes and degrees of overdispersion (though it is possible to model 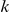 as function of other variables [19], [20]). The second approach assumes that hosts randomly sample parasites from their environment, which would result in a Poisson distribution of burdens, but that the sampling rate (i.e., the expected burden) varies among hosts [1], [16], [21]. When the sampling rate of the Poisson is gamma distributed, the marginal distribution of burdens is negative binomial [1], [16], [22]. Variation in the sampling rate among hosts therefore causes overdispersion. In contrast to the regression approach, the Poisson-gamma mixture directly results in a NBD of burdens and the aggregation parameter, 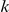, can be expressed in terms of the parameters of the sampling rate distribution [23]. Unfortunately, the variation in sampling rates that drives aggregation is typically unobserved, and thus the causes of this variation are not identified.

Empirical studies of tick aggregation have focused heavily on identifying intrinsic host characteristics that explain observed tick burdens via the regression approach. The rationale is that *a priori* identification of hosts likely to have large burdens could lead to targeted and highly effective control efforts. Unfortunately, these efforts have, so far, produced equivocal results, with few consistent factors emerging across different studies, systems, sites, and years. Males often have greater burdens than females (e.g., on *A. flavicollis*, [11]), but two recent studies have shown that sex is just one of myriad host and environmental variables that each explain a small portion of the variability in burdens on several rodent species [12], [14]. Even a study that explicitly linked activity/exploration phenotypes of Siberian chipmunks (*Tamias sibiricus*) to tick burdens found that many variables, including their interactions, were significantly associated with burdens [13]. It is still not clear from these studies how much of the observed aggregation of tick burdens is due to variation in susceptibility among hosts, and how much is due to extrinsic factors such as variation in questing tick densities among host home ranges. If tick burdens are driven primarily by random, extrinsic factors, control efforts focusing on identifying the most susceptible hosts via host characteristics may be doomed to fail.

We have two main goals in this paper. First, we introduce a general framework for understanding the distribution of parasites on hosts. The framework consists of a simple and flexible mechanistic model of parasite accumulation that could be easily tailored to a wide range of host-parasite systems, and an explicit consideration of how variability among hosts enters into the parasite accumulation process. Our framework can be understood as an extension of the Poisson-gamma mixture model widely invoked to explain negative binomial parasite distributions. Second, we use a hierarchical Bayesian approach to couple our model to the best available data on blacklegged ticks and white-footed mice to quantify the degree to which random variation in tick density among mouse home ranges affects the overdispersion of tick burdens on mice.

## Methods

As mentioned above, the key weakness of using the Poisson-gamma mixture to model parasite burden distributions is that sampling rate variation is usually unobserved. Because of this, it is often not possible to identify the causes of aggregation with burden data alone. We seek here to extend the Poisson-gamma mixture framework by linking variation in the sampling rate among hosts to an underlying model of parasite accumulation. In other words, we aim to write the NBD and its aggregation parameter, 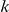, in terms of an accumulation model and associated sources of variability among individuals. It will then be possible to use burden data in combination with other types of data that can speak directly to the accumulation process. In our empirical example, we focus on a key extrinsic factor, spatial variation in larval blacklegged tick (*I. scapularis*) density, to quantify its contribution to aggregation relative to that of intrinsic differences in susceptibility among white-footed mice (*Peromyscus leucopus*).

We first derive a simple model relating host movement and tick density to the expected burden, or sampling rate, 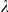, on an individual host. We assume that the realized tick burden on a host, 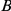, is Poisson distributed with rate parameter 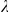. We then consider how variation in 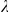 among hosts gives rise to an approximately negative binomial distribution of burdens. Table 1 summarizes the symbols and notation we use throughout the paper.

**Table 1:** A summary of the notation used in the paper.

Symbol	Description	Units (if applicable)	Possible subscripts
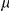	Mean of burden dist.	num.	Cls, Rnd, Cor
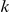	Aggregation param. of burden dist.		Cls, Rnd, Cor
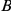	Larval tick burden	num.	
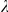	Expected tick burden (a host's “sampling rate”)	num.	
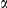	Tick accumulation constant	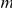	
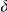	Tick loss constant	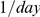	
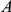	Host home range area	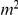	
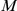	Host movement rate	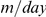	
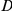	Within home range tick density	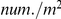	
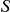	Susceptibility factor	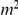	
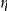	Gamma dist. shape param.		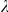 , 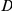 , 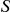
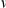	Gamma dist. scale param.		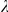 , 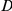 , 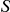
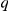	Extrinsic factor index		

### A simple tick accumulation model

We assume that each host occupies a home range characterized by its area, 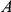, and parasite density, 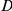. Hosts move within their home ranges and encounter ticks as they do so. The the per day rate at which a host encounters and picks up parasites should then be proportional to the product of the distance it moves per day, 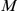, and the density of questing larvae, 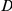, in its home range.

We further assume that each home range can be characterized by the average parasite density the host experiences in that home range, and that this density remains constant during the study period. This later assumption implies that the removal and feeding of parasites by hosts and by parasite mortality are insignificant compared to the number of questing parasites available in a home range, at least over a short period of time. This assumption is reasonable for our blacklegged tick/white-footed mouse example, given that densities of questing nymphs and larvae, as well as burdens on mice, remain high for several weeks during the peak of the season [12], [24].

Lastly, we assume that the parasites feeding on a host drop off after successfully feeding or are removed (e.g., by grooming or host immune responses) at a constant rate, 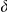, independent of the density of ticks on the host (although it is possible to modify this assumption). The expected tick burden, 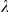, on a host at time 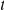 is therefore determined by the rates at which parasites are picked up and lost, or
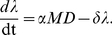
(1)Notice that the accumulation constant, 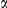, could be broken into a number of individual constants including the width of area “sampled” by the host, the probability that given an encounter, the parasite attaches to the host, etc. These constants all enter as a product whose individual components are not separately estimable from the data at hand, and so we lump them together. Lumping these factors into a single constant is standard practice in models that have an encounter or accumulation term, such as predator-prey models (e.g., see derivation of the predator functional response curves in [25]). If more detailed data were available, the components of 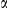 could be kept separate.

Assuming that parasite burdens at the time of sampling have reached their equilibrium (again, for our example system, the seasonal “peaks” in tick burdens last for several weeks) [12], we focus on the stationary solution of equation (1):
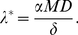
(2)


For notational convenience, we will refer hereafter to the equilibrium sampling rate simply as 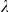, dropping the 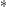 superscript. Again, if data on host movement or the components of 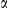 are available, these factors could be kept separate, and the steps below could then be performed on this expanded model. Focusing on our empirical example, we assume that 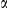, 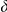, and 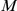 are intrinsic to the individual, while variation in 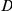 is extrinsic to the individual. Defining 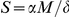, collects all intrinsic components of the model into a single “susceptibility” factor. While it could be argued that movement rates are determined by extrinsic factors such as resource densities, it is equally true that the ability of an individual to occupy and maintain a home range with a given level of resources depends on intrinsic characteristics, such as sex, age class, and the animal's condition. In any case, by focusing on parasite densities, 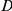, which is clearly extrinsic to the host, and letting these other factors be included in the variable 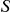, we are being conservative about the importance of extrinsic factors.

### The approximate distribution of tick burdens

Both 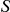 and 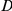 will vary among hosts and will thus be considered random variables. As both are continuous, must be non-negative, and could conceivably assume a range of different distributional shapes, we assume they are gamma distributed. We denote shape parameters of gamma distributions by 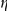 and scale parameters by 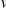. Thus, the gamma distribution of 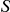 is characterized by 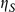 and 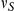, while that of 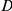 is parameterized by 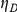 and 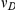.

When derived as a Poisson-gamma mixture, the probability mass function of the NBD can be written in terms of the parameters of the gamma distribution of sampling rates [23], yielding
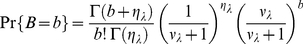
(3)where 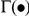 is the Euler gamma function, and 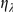 and 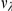 are the shape and scale parameters, respectively, of the sampling rate distribution.

Equation (2) shows that 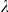 is the product of 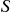 and 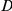. In appendix S1, we show via simulation that a gamma distribution provides a good approximation of the distribution of the product 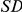. We then use this fact to derive an approximation that allows us to write the parameters of the approximate gamma distribution of 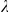 in terms of 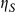, 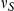, 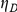, and 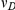. The resulting approximate expressions for the shape and scale parameters of the rate distribution are (appendix S1)
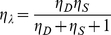
(4)and
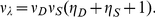
(5)


Substituting equations (4) and (5) into equation (3), we obtain an approximation for the distribution of parasite burdens over hosts in terms of the accumulation model and associated sources of variability. The mean of the burden distribution is
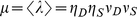
(6)and the aggregation parameter of the burden distribution is 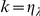. In other words, the degree of aggregation in tick burdens is determined by the shape parameter of the gamma distribution that describes how accumulation rates vary among individual hosts. The rate distribution shape parameter is, in turn, a function of 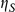 and 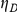. Thus, this approximation links lower-level processes governing vector accumulation, which are potentially measurable, to the degree of aggregation in vector burdens on hosts.

Focusing on our blacklegged tick example, we can now develop an index that quantifies the contribution of variation in questing larval density among host home ranges, 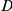, to the observed value of 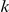. The limit of the expression for 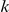 as 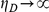 (i.e., as the effect of variation in larval density goes away) is simply 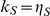. In this limit, equations (3), (4), (5), and (6) are exact (i.e., the rate parameter distribution is a gamma) and variation among individuals in sampling rate is driven entirely by differential susceptibility. The ratio 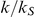 will then be 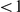 as long as 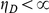, and the quantity 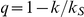 is a measure of the degree to which the estimated value of 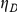 reduces the value of 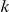 conditional on the value of 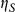. In other words, 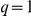 when the aggregation of vectors on hosts is dominated by differences in vector densities among home ranges and is zero when the aggregation is due entirely to differences in individual susceptibility. Writing 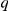 in terms of 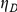 and 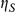, we obtain
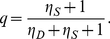
(7)Checking the limit behavior of 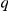, we see that 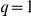 when 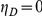, and 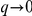 when 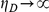, as expected.

### Empirical data

We now show how the above-described extended Poisson-gamma mixture can be combined with empirical data to tease apart the contribution of extrinsic and intrinsic factors on the larval tick burdens of white-footed mice. We used two years of data (1999 and 2004) from two of the six 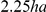 permanent small mammal trapping grids (GC and TX) in the oak and maple dominated forests tracts of the Cary Institute of Ecosystem Studies (CIES) that have been trapped for 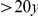 by R.S. Ostfeld and colleagues. We chose these years and grids because they offered the most observations of larval burdens and mouse home range sizes and densities of questing larvae, used to estimate 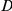. A more detailed description of the trapping methods can be found elsewhere [26].

Questing activities and larval burdens are highly seasonal [24], showing fairly distinct, but broad peaks in the late summer/early fall (mid- to late-August into early September). We therefore restricted our analyses to the data collected during these peaks, as visually identified. In addition, individual mice were often captured several times (this being a mark-recapture study). In order to avoid multiple non-independent measurements of tick burdens, we selected at random only one observation per individual mouse. *Ixodes scapularis* were counted on each mouses' head and ears, and these counts are highly correlated with whole-body larval burdens (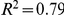) [26].

Densities of host-seeking, or “questing”, larvae at a site were estimated using standard drag cloth methods [27] along 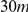 transects, so the grain of our tick density data is 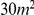. Dragging was done several times during the expected peaks of larval activity, but the actual dates of dragging were inconsistent between years and trapping grids. We therefore restricted our analyses to the three or four transects that coincided with and straddled the peaks of questing larvae densities and of larval burdens.

Ideally, we would have data on larval tick densities across the home ranges of individual mice, or at least at the scale of mouse home ranges. As with every other study we are aware of, our tick density data are not paired with individual mice and estimated mouse home range areas are generally much larger than the 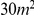 tick drags (see appendix S2). To deal with this issue, we upscaled the density data to the home range sizes using two assumptions about the spatial correlation among 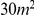 samples, which bracket the range of possibilities. The upscaling proceeds by selecting a random home range size from the home range area distribution (appendix S2). This area is then “filled” 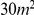 at a time from the distribution of 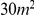 larval drags (appendix S2). The larval drag transects are widely spaced within the trapping grids. Filling each home range with random samples from the tick density distribution corresponds to one extreme where there is no short distance correlation in tick densities (hereafter Rnd). Thus the filled home ranges all tend towards the overall mean tick density and among home range differences are at their minimum. The other extreme, perfect short distance correlation in tick densities (hereafter Cor), can be obtained by taking a single random sample from the larval drag distribution and multiplying it by 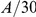, where 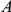 is the area of the focal home range. In this case, the large degree of heterogeneity observed among 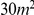 drags is preserved at the scale of entire home ranges. For each grid/year combination, this procedure was repeated 10000 times for each of the Rnd and Cor assumptions. Finally, random samples of 15 areas (matching the smallest actual sample size involved, that of larval drags for each grid year combination) were drawn from the Rnd and Cor distributions for each grid/year combination. This resulted in 8 datasets: 2 grids×2 years×2 density upscaling assumptions.

### Hierarchical Bayesian parameterization of the accumulation model

**Figure 1:**
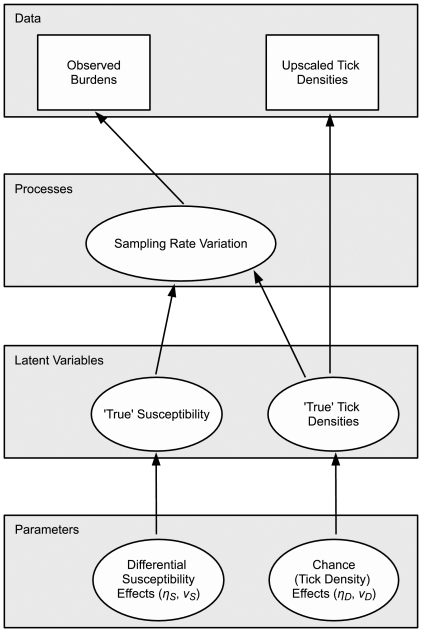
Hierarchical Bayesian model structure of the tick accumulation model. Gray boxes identify the levels in the hierarchy, white boxes represent data, and white ovals represent low-level model elements. Arrows show the relationships among model elements.

We employed a hierarchical Bayesian (HB) framework to fit our accumulation model to the larval burden and the upscaled larval density datasets (Fig. 1) [28], [29]. The framework includes two latent variables–“true susceptibility” and “true tick density”–to account for the facts that: 1) susceptibility is not directly observed, and 2) “observations” of upscaled larval tick densities cannot be directly paired with observations of tick burdens. The overall likelihood is thus a product of the two conditionally independent likelihoods of the data sources (burdens and upscaled densities), conditioned on the values of the latent variables (Fig. 1).

We used noninformative (uniform) priors for all four model parameters (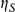, 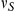, 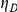, and 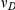) on the four Rnd datasets. For the four Cor datasets, a weakly informative half-Cauchy prior [29], [30] was used on 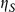 to achieve convergence (see appendix S3 for explanation), while uniform priors were used for the other three parameters. Though this prior introduces a slight bias in the results in favor of increasing the apparent contribution of differential susceptibility, its effect on our qualitative results is negligible: Upscaled tick densities account for most of the aggregation in burdens in the Cor datasets (table 2).

**Table 2:** Grid-specific Bayesian mean estimates for the accumulation model parameters.

	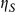	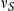	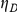	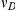	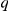
GC 1999 Rnd	1.89	11.71	9.50	0.17	0.27
	(1.31, 2.89)	(6.82, 17.72)	(3.47, 22.24)	(0.06, 0.38)	(0.10, 0.52)
GC 1999 Cor	5.24	4.89	1.99	0.75	0.73
	(2.27, 16.14)	(1.08, 9.90)	(1.38, 2.86)	(0.45, 1.28)	(0.56, 0.93)
GC 2004 Rnd	1.26	8.15	9.03	0.22	0.23
	(0.93, 1.82)	(5.00, 11.53)	(3.04, 18.81)	(0.08, 0.54)	(0.10, 0.50)
GC 2004 Cor	2.91	4.16	2.10	0.93	0.62
	(1.28, 9.32)	(0.92, 8.28)	(1.18, 3.63)	(0.44, 1.78)	(0.41, 0.88)
TX 1999 Rnd	3.66	11.95	3.64	0.18	0.54
	(1.47, 13.35)	(2.31, 24.07)	(1.66, 6.86)	(0.08, 0.37)	(0.29, 0.90)
TX 1999 Cor	19.52	2.02	1.21	0.70	0.92
	(4.06, 68.40)	(0.33, 6.02)	(0.92, 1.56)	(0.40, 1.16)	(0.79, 0.99)
TX 2004 Rnd	1.42	14.65	25.92	0.03	0.10
	(1.01, 1.97)	(9.54, 21.67)	(9.45, 49.40)	(0.02, 0.08)	(0.04, 0.23)
TX 2004 Cor	7.34	4.03	1.73	0.52	0.77
	(1.98, 32.07)	(0.48, 9.93)	(1.15, 2.60)	(0.29, 0.90)	(0.55, 0.96)

95% credible intervals are in parentheses below the point estimates. The sample sizes for the burden datasets are 132, 165, 96, and 91 for GC 1999, GC 2004, TX 1999, and TX 2004, respectively. A sample size of 15 was used for all upscaled density datasets.

We implemented this approach via MCMC sampling in WinBugs 1.4 [31]. The WinBugs code including the priors is listed in appendix S3. All analyses except for TX 1999 Cor employed a 70,000 iteration burn-in period followed by 30,000 iterations of which 5000 were kept as samples from the posterior distribution. A longer burn-in period of 150,000 iterations was used for TX 1999 Cor. For each dataset, we ran three chains started from widely spaced initial conditions. We used 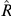, the Gelman-Rubin statistic [32], to verify convergence was achieved (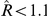 for all model parameters). Finally, we used posterior predictive simulations to check the fit of the models to the burden data and to propagate uncertainty in model parameters through to summary quantities that are functions of model parameters (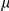, 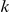 and 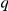).

### Comparison of the accumulation model to the classical NBD

The NBD as commonly used in parasitology and ecology is parameterized by its mean, 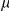, and aggregation parameter, 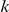, which are estimated from count (e.g., burden) data via maximum likelihood [1], [22], [23]. Our accumulation model is parameterized in terms of the distributions of 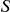 (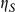 and 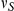) and 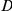 (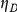 and 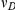), but equations (4) and (6) allow us to calculate the aggregation parameter, 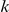, and the mean, 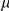, respectively, of the burden distribution that results from our Bayesian fit of the accumulation model to each dataset. Thus, it is possible to compare our accumulation model, which is fit using upscaled tick density data in addition burden data, to the classical NBD, which is fit using only burden data. This comparison serves two purposes. The first is as a consistency check of the new accumulation model in that the model should provide similar values of 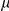 and 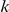 as those obtained by fitting the classical NBD via maximum likelihood. The second purpose is to examine how the two upscaling assumptions affect the degree of aggregation, as measured by 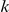, relative to that obtained by fitting the empirical NBD. We use the subscript Cls to refer to the classical NBD, and the subscripts Rnd and Cor to refer to the accumulation model fitted to the Rnd and Cor upscaled density datasets, respectively.

## Results

**Figure 2:**
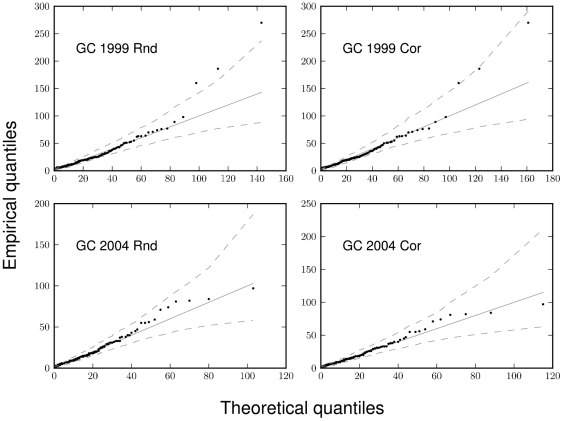
Bayesian fits of the model to the four GC grid datasets, as visualized with quantile-quantile plots. The “expected” distribution (solid lines) under the fitted accumulation model, as well as the 95% credible regions (dashed lines) around the predicted line, were generated via posterior predictive simulations.

**Figure 3:**
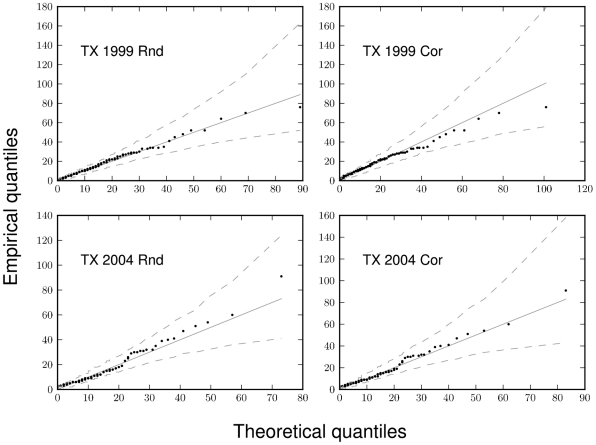
Bayesian fits of the model to the four TX grid datasets, as visualized with quantile-quantile plots. The “expected” distribution (solid lines) under the fitted accumulation model, as well as the 95% credible regions (dashed lines) around the predicted line, were generated via posterior predictive simulations.

The parameterized HB models successfully described the observed distributions of larval blacklegged ticks on white-footed mice (Figs. 2 and 3). The expected burden distribution under the fitted model and the 95% credible regions in these figures are obtained by posterior predictive simulation. The model fits well in most cases, with some disagreement in the upper quantiles for GC 1999 Rnd, and to a lesser extent, for GC 1999 Cor (Fig. 2). Table 2 presents Bayesian posterior means and 95% credible intervals for accumulation model parameters.

A key strength of our analytical approximation of the burden distribution is that it allows us to directly examine the factors that drive aggregation. Equation (4) shows that 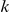 depends only on the shape parameters of the distributions of 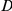 and 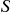. The effect of each variable on 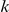 goes away as its shape parameter becomes large and its distribution becomes symmetrical. Thus skewness in each component distribution, as indicated by a small shape parameter value, translates into aggregation in the overall distribution of burdens. This can be seen by examining how the 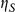 and 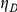 point estimates change between the Rnd and Cor versions of each site/year dataset (Table 2). In all but the TX 1999 Rnd case, where 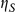 and 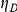 are essentially equal, 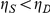 for the Rnd datasets, and 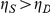 for the Cor datasets.

Point estimates of 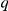, the degree to which the aggregation in tick burdens is driven by variation in questing larval density, are higher in the Cor datasets (those in which tick distributions are spatially autocorrelated) than in the Rnd datasets (those without spatial autocorrelation) (Table 2). This indicates that strongly skewed tick density distributions (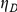 small) can account for most of the aggregation observed in the burden data. Even in the Rnd datasets, where tick densities in different hosts' home ranges are more similar, point estimates for 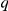 can be as high as 0.54 (TX 1999 Rnd), and are never less than 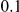, indicating that variability in the tick density experienced by different mice can still play a substantial role in explaining observed burdens.

Table 3 compares the posterior predictive mean values of the burden distribution mean and aggregation parameter under the fitted accumulation model for both the Rnd (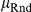 and 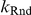) and Cor (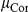 and 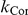) datasets to their empirical counterparts (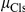 and 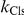). The point and interval estimates obtained by the different methods are generally similar, further demonstrating the consistency between the accumulation model and observed data. Though there is substantial overlap between the Rnd and Cor datasets, the Cor datasets produce somewhat lower values of 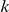 than those estimated directly from the burden data, suggesting that such strong spatial correlation in tick density introduces too much aggregation in burdens. This tendency suggests that, at least in extreme scenarios, variation in the tick density experienced by different individuals can be more than enough to account for the observed aggregation in parasite burdens.

**Table 3:** The upper section contains grid-specific maximum likelihood estimates of the mean (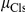) and aggregation parameter (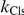) obtained by directly fitting the classical NBD to the larval burden data via maximum likelihood.

	GC 1999	GC 2004	TX 1999	TX 2004
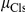	28.16	15.87	17.41	14.59
	(23.97, 32.36)	(13.43, 18.31)	(14.45, 20.38)	(11.86, 17.32)
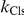	1.38	1.05	1.50	1.31
	(1.06, 1.69)	(0.82, 1.28)	(1.06, 1.93)	(0.92, 1.71)
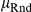	27.61	15.91	17.76	14.71
	(23.86, 32.64)	(13.55, 18.69)	(14.76, 21.74)	(12.14, 17.80)
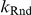	1.34	0.96	1.29	1.27
	(1.01, 1.70)	(0.74, 1.20)	(0.91, 1.77)	(0.935, 1.71)
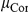	27.83	16.07	18.23	14.83
	(23.69, 32.82)	(13.77, 18.89)	(14.66, 22.53)	(11.99, 18.37)
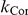	1.16	0.87	1.02	1.06
	(0.87, 1.50)	(0.66, 1.15)	(0.72, 1.33)	(0.73, 1.48)

Wald-type 95% confidence intervals are in parentheses below the MLEs. The bottom section presents the corresponding Bayesian posterior predictive means and 95% posterior predictive intervals for the Rnd (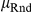 and 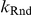) and Cor (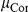 and 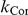) datasets.

## Discussion

We have developed an extension of the classical Poisson-gamma mixture model of overdispersed parasite burdens by linking among-host variation in parasite sampling rate to a mechanistic model of parasite accumulation. The key idea that the distribution of parasite sampling rates among hosts can be related to lower-level parasite encounter and accumulation processes is very general and should apply to many host/macroparasite systems. While certain details of the accumulation process will likely be system specific, the formulation of the accumulation model is flexible and can be tailored to such details when necessary. When embedded in a hierarchical Bayesian statistical framework, our model allows multiple sources of information, acting on different hierarchal levels, to be coherently integrated. The parameters of the distribution of parasite burdens can be written in terms of the components of the accumulation model and thus be linked to lower-level processes, and uncertainty in model parameters can be propagated through to quantities that are functions of model parameters (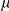, 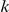, and 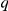). Our framework differs from other models of macroparasite burdens in that it describes the shape of the distribution of burdens as a function of a biologically relevant parameters rather than simply treating the overdispersion parameter, 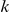, as a nuisance parameter, as in regression-based approaches, or leaving the variation in sampling rates among hosts unexplained, as in traditional applications of the Poisson-gamma mixture.

With only four free parameters, our model is able to reproduce the observed distribution of blacklegged tick burdens on white-footed mice in several places and times. Moreover, it provides a novel way to separate the contribution of intrinsic factors affecting parasite aggregation from that of extrinsic factors such as spatial variation in parasite density. The burden data provide strong information about 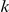, while the upscaled tick density data provide information about 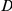, both directly and indirectly through the accumulation model (Fig. 1). Overdispersion in burdens that cannot be accounted for by 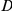 is absorbed by the latent variable 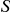 and is thus attributed to differential susceptibility. Additional information, such as data on individual rates of host movement or grooming could easily be accommodated within our framework to provide more precise estimates of the parameters governing 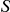.

We have identified patchiness in the spatial distribution of questing tick density as a key factor in explaining observed burden distributions. Highly patchy distributions imply strong short-distance correlation in tick density, meaning adjacent areas will likely have similar tick densities on small spatial scales. Though we could not directly quantify this correlation with available data, we based our analyses on two extreme assumptions (no correlation and perfect correlation) that bracket the range of possibilities. In the Rnd extreme, where tick densities among home ranges tended toward the site-level mean, variation in tick densities was still important in some locations and years (e.g., TX 1999 Rnd), while it was less of a factor in others (e.g., TX 2004 Rnd). Importantly, the influence of this extrinsic factor did not completely disappear in any of the cases considered under the Rnd assumption (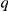 values ranged from 0.1 to 0.54). Examining the other extreme (Cor), where the differences in tick densities among home ranges were most pronounced, we see that there is a tendency toward slightly too much aggregation, as demonstrated by the posterior predictive mean 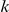 values in table 3. This implies that highly patchy tick spatial distributions can account for most or all of the aggregation observed in tick burden distributions. Furthermore, questing tick density had a strong effect on observed 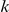 values at all grid/year combinations examined under the Cor assumption. As questing larvae distributions are known to be highly patchy [33], [34], we argue that the degree of correlation will likely fall closer to the Cor extreme than to the Rnd extreme.

Our results suggest that the often extreme differences in individual tick burdens we observe are not solely or, in many cases, even mostly caused by intrinsic differences in individual susceptibility due, for instance, to sex or life history stage. These differences in burdens can instead be explained primarily by random differences in the densities of questing ticks experienced by different hosts. As questing tick densities become more variable among home ranges, so ticks become more aggregated on a relatively small proportion of hosts. In other words, our results imply that some mice may have extremely large tick burdens simply because of bad luck; their home ranges happen to overlap with areas of high tick density.

Spatial variation in questing larval density is very clearly a product of random processes. Gravid *I. scapularis* drop to the ground after feeding to repletion on their blood meal host, usually a deer or other larger bodied mammal, wherever that may be, and lay eggs close to where they fall [24]. The adult females and resulting larvae move no more than a meter or two while questing for a host [35]. There is no evidence, to the best of our knowledge, that gravid females choose where to drop to the ground. If there is a deterministic aspect to local questing larval densities, it is that some locations may be more favorable for larval hatching and survival [36]. Our results highlight the importance of quantifying questing tick density within each host home range. The availability of such data would improve our ability to pin down the mechanisms driving the aggregation of vectors on hosts. We are currently attempting to directly quantify the relationship between home-range-scale larval tick density and host body burden in the blacklegged tick/white-footed mouse system. Such a dataset will facilitate a direct test of our main empirical conclusion here. Quantifying variability in movement rates among hosts would further refine our understanding of the mechanisms governing parasite accumulation.

Our main empirical result, that variation in tick densities among home ranges can strongly affect tick burdens, is, one the one hand, not surprising. It is well known that tick spatial distributions are patchy on relatively small scales [33], [34], and it is logical to expect that this variability will affect tick burdens on hosts. On the other hand, the focus in the literature has very clearly been on trying to identify *a priori* biological characteristics that reliably predict parasite burdens [11], [12], [14]. This search assumes that host-related factors account for the majority of the variation in observed burdens. Our results cast substantial doubt on this assumption, and suggest that more effort should be spent on testing it and on quantifying the contribution of random, extrinsic factors. As much of the variation in tick burdens could potentially be explained by largely unpredictable, small-scale variation in the density of questing ticks, our results imply that it may be impossible to predict *a priori* the type(s) of individuals that will accumulate the largest burdens, and thus make the greatest contribution to disease transmission. Management strategies that assume such an *a priori* determination of heavily burdened individuals is possible may therefore prove ineffective and may waste limited management resources. Instead, management strategies that focus on finding and mitigating concentrations of questing larval ticks might inhibit heavy larval burdens on mice and the resulting production of numerous infected nymphs.

## Supporting Information

Appendix S1 Approximation of the sampling rate distribution.
(PDF)

Appendix S2 Home range sizes and upscaled larval density.
(PDF)

Appendix S3 WinBUGS code and information about priors.
(PDF)
