# Supplementary material for: Partitioning the Aggregation of Parasites on Hosts into Intrinsic and Extrinsic Components via an Extended Poisson-Gamma Mixture Model
Source: PLoS One. 2011 Dec 22;6(12):e29215. doi: 10.1371/journal.pone.0029215 (PMC3245270; doi:10.1371/journal.pone.0029215)
Supplement: Appendix S1 — Approximation of the sampling rate distribution. (PDF) [file pone.0029215.s001.pdf]

Appendix S1: Approximation of the sampling rate distribution

First, we show via simulation that a gamma distribution provides a good approximation of the distribution of the sampling rate, λ, at the values of the accumulation model parameters estimated from the data. We then use this result to derive an analytical approximation that allows us to write the gamma rate distribution parameters in terms of η_D, ν_D, η_S, and ν_S. The simulation of λ proceeds by first choosing a set of accumulation model parameter values, and then drawing K observations each from the distributions of S and D, multiplying each pair of values thus obtained, and storing the results. We then fit a gamma distribution to the simulated value via maximum likelihood, and compared the fitted gamma to the simulated values using a qq-plot (Fig. 1). The 95% confidence bands in figure 1 are obtained assuming that the parameters of the fitted gamma are known without error (i.e., treating it as the true distribution and the simulated values as a sample to be compared to it). These confidence bands are thus narrower than would be the case had we acknowledged this uncertainty and this comparison is thus conservative. Figure 1 demonstrates that the gamma provides a reasonable approximation of the distribution of lambda across the extremes of the parameter values shown in table 2 in the main text.

We now want to obtain the parameters η_λ and ν_λ of the approximate gamma distribution of sampling rates. We do this by first finding the mean and variance of the quantity $\lambda = SD$ when both S and D are gamma distributed random variables. Though we do not know the full distribution of λ, with exact expressions for its mean and variance in hand, we can approximate it by fitting a gamma distribution to it via the method of moments.

The mean of a gamma distribution with parameters η and ν is $\eta\nu$, while the variance is $\eta\nu^2$. Recalling basic results from probability theory, the mean of the product of two uncorrelated random variables is simply the product of the means, so

$$\langle\lambda\rangle = \eta_D\eta_S\nu_D\nu_S. \tag{1}$$

where the angle bracket notation indicates a mean. The variance is given by

$$\mathrm{Var}(\lambda) = \mathrm{Var}(D)\langle S\rangle^2 + \mathrm{Var}(S)\langle D\rangle^2 + \mathrm{Var}(D)\mathrm{Var}(S) = \eta_D\eta_S\nu_D^2\nu_S^2(\eta_D + \eta_S + 1). \tag{2}$$

Finding distributions of products of random variables is in general a difficult task and we will not attempt it here. Instead, we can approximate the distribution of λ with a gamma distribution using the method of moments. A key feature of the gamma distribution here is that it has simple moments and thus simple moment estimators. The shape and scale parameters of the best-fit gamma rate distribution are given by

$$\eta_\lambda = \frac{\langle\lambda\rangle^2}{\mathrm{Var}(\lambda)} \quad \text{and} \quad \nu_\lambda = \frac{\mathrm{Var}(\lambda)}{\langle\lambda\rangle}, \tag{3}$$

which, after substituting equation (1) for $\langle\lambda\rangle$ and equation (2) for $\mathrm{Var}(\lambda)$ and simplifying, are given in the main text as equations (4) and (5), respectively.

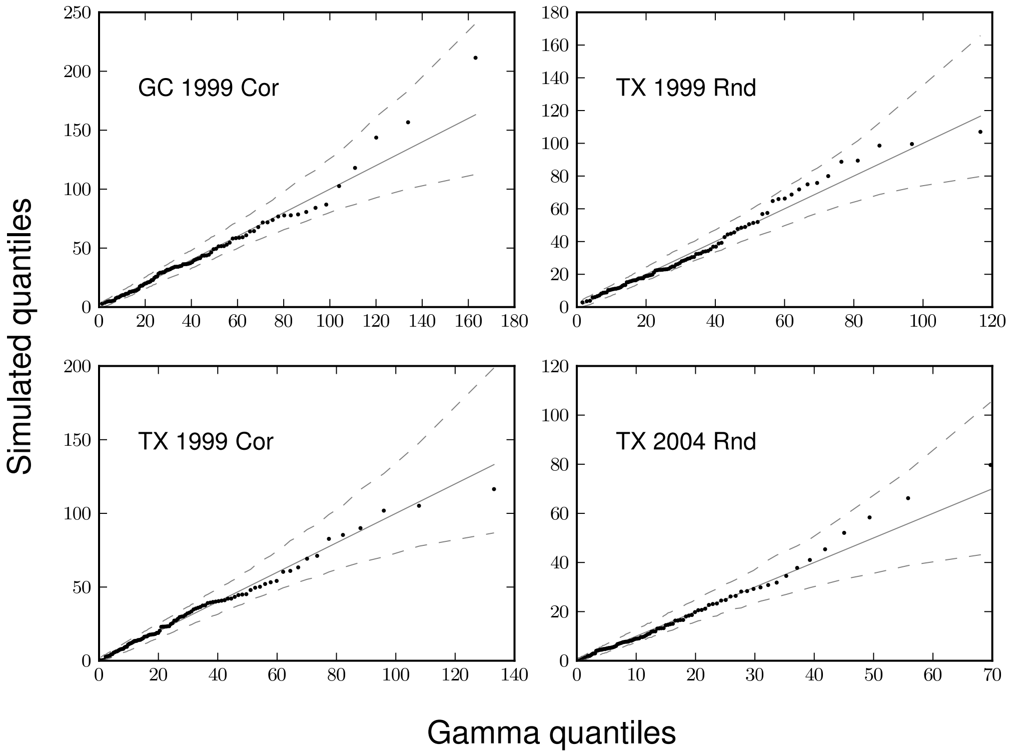

Figure 1: Graphical check of the approximation of the rate parameter distribution with a gamma distribution. The solid line is the best-fit gamma and the dashed lines represent 95% confidence bands. The sample size used throughout was $K = 100$, similar to the sample sizes of the burden datasets.
