# Supplementary material for: Partitioning the Aggregation of Parasites on Hosts into Intrinsic and Extrinsic Components via an Extended Poisson-Gamma Mixture Model
Source: PLoS One. 2011 Dec 22;6(12):e29215. doi: 10.1371/journal.pone.0029215 (PMC3245270; doi:10.1371/journal.pone.0029215)
Supplement: Appendix S2 — Home range sizes and upscaled larval density. (PDF) [file pone.0029215.s002.pdf]

Appendix S2: Home range sizes and upscaled larval density

In order scale the density of questing ticks to the level that a mouse would encounter we needed to know how large mouse home ranges are and how they are distributed. We estimated the home range sizes of individual mice as the minimum convex polygon that captured 95% of an individual's capture locations on the grid in a given year using the mcp.habitat() function in package adehabitat [1] for R [2]. Since estimates of home range size will necessarily increase with the number of captures, at least initially, we only used individuals with at least 10 captures in a year. While the average home range size does increase from ca. $500m^2$ with 10 captures to ca. $1000m^2$ with 18 captures, this difference is small compared to the variation in home range size among individuals with the same number of captures (not shown). When mouse densities are high estimates of home range size based on trapping are very close to estimates based on radiotelemetry [3]. The distributions of home range sizes were reasonably described by gamma distributions (parameters in table 1).

We also fit gamma distributions to the $30m^2$ larval drag data for each grid/year combination, and the results are shown in table 1. We then sampled from these fitted distributions of mouse home range areas and larval tick densities to implement the upscaling procedure described in the main text. For each grid/year combination, the upscaling procedure resulted in a distribution of tick densities for each of the Rnd and Cor assumptions. The parameters of these upscaled density distributions are given in table 2.

Table 1: Maximum likelihood estimates of gamma distribution parameters and associated goodness-of-fit tests for the larval drag data (upper section) and the mouse home range area data (lower section). Standard errors are in parentheses.

		GC 1999	GC 2004	TX 1999	TX 2004
LD	shape (η)	1.25 (0.41)	1.80 (0.61)	0.60 (0.18)	0.86 (0.27)
	scale (ν)	34.19 (13.72)	24.70 (9.56)	30.17 (13.65)	24.30 (10.25)
	χ^2	3.77	4.46	2.22	3.62
	p	0.15	0.11	0.14	0.06
HR	shape (η)	3.07 (0.71)	1.89 (0.45)	1.86 (0.47)	1.86 (0.39)
	scale (ν)	146.87 (39.99)	184.31 (54.27)	208.64 (66.86)	416.63 (143.68)
	χ^2	6.84	2.04	2.96	4.99
	p	0.08	0.36	0.09	0.08

Table 2: Parameters describing the gamma distributions of upscaled tick density under both the Rnd (upper section) and Cor (lower section) upscaling assumptions.

		GC 1999	GC 2004	TX 1999	TX 2004
Rnd	shape (η)	13.79	13.52	4.88	11.99
	scale (ν)	0.10	0.11	0.12	0.06
Cor	shape (η)	1.27	1.78	0.60	0.87
	scale (ν)	1.11	0.83	1.00	0.80

References

[1] Calenge C (2006) The package "adehabitat" for the r software: a tool for the analysis of space and habitat use by animals. Ecological Modelling 197: 516–519.

[2] R Development Core Team (2009) R: A language and environment for statistical computing. Vienna, Austria: R Foundation for Statistical Computing.

[3] Ribble DO, Wurtz AE, McConnell EK, Buegge JJ, Welch KC (2002) A comparison of home ranges of two species of *Peromyscus* using trapping and radiotelemetry data. Journal of Mammalogy 83: 260-266.
