# Supplementary material for: Partitioning the Aggregation of Parasites on Hosts into Intrinsic and Extrinsic Components via an Extended Poisson-Gamma Mixture Model
Source: PLoS One. 2011 Dec 22;6(12):e29215. doi: 10.1371/journal.pone.0029215 (PMC3245270; doi:10.1371/journal.pone.0029215)
Supplement: Appendix S3 — WinBUGS code and information about priors. (PDF) [file pone.0029215.s003.pdf]

Appendix S3: WinBUGS code and information about priors

Here, we describe the reasons for using an informative prior on η_S for the Cor assumption. For the Cor datasets, the shape parameter of the upscaled density distribution, η_D is small (appendix S2, table 2). Examining equation (4) in the main text reveals that a small value of η_D will lead to a small value of k. As the η_D values in the Cor datasets are sufficiently small to account for most of the aggregation in the burden data, corresponding estimates of η_S will tend to be large. However, the effect of η_S on k vanishes as it becomes large, so that further increases in η_S have essentially no effect on model fit. Though the Cor datasets support values of η_S that are larger than those of η_D, there is little information available to pin down a specific value. The half-Cauchy prior exerts a slight pull on η_S towards the origin, stopping it from wandering around at arbitrarily large values.

For all of the Cor datasets except TX 1999 Cor, the scale parameter, σ, of the half-Cauchy prior was 25 [1]. For TX 1999 Cor, a stronger half-Cauchy prior on η_S ($\sigma = 15$) was necessary to ensure convergence. For all uniform prior densities we checked that the ranges chosen for the priors were not influencing the results.

The following listing shows the WinBugs code, including specification of the priors, used to fit the model to the burden data, B, and upscaled larval density data, D.

```
model
{

#Loop over observed burdens
for(i in 1:NB)
 {

  #Upscaled tick density component, D, of the accumulation model
  vdInv[i] <- 1/vd
  dd[i] ~ dgamma(nd, vdInv[i])

  #Susceptibility component, S, of the accumulation model
  vsInv[i] <- 1/vs
  ss[i] ~ dgamma(ns, vsInv[i])

  #The rate parameter of the Poisson is the product of the accumulation
  #model components
  lambda[i] <- dd[i] * ss[i]

  #The burden data are then distributed as a Poisson with this
  #rate parameter
  B[i] ~ dpois(lambda[i])
 }

#The upscaled tick density data fit to the gamma
for(i in 1:ND)
{
  vdInv[NB+ i] <- 1/vd
  D[i] ~ dgamma(nd, vdInv[NB + i])
}
```

```
### Prior distributions for the four model parameters

#Uniform prior on nu_S
vs ~ dunif(0.001, 250)

#Uniform prior eta_S for the Rnd datasets
#ns ~ dunif(0.01, 200)

#Half Cauchy prior on eta_S for the Cor datasets
#Scale parameter is 25, so precision of xiNs = 1/25^2 = 0.0016
xiNs ~ dnorm(0, 0.0016)I(0.01,)
chSqNs ~ dgamma(0.5,0.5)
ns <- xiNs/sqrt(chSqNs)

#Uniform priors on eta_D and nu_D
nd ~ dunif(0.01, 60)
vd ~ dunif(0.001, 20)

}
```

References

[1] Gelman A (2006) Prior distributions for variance parameters in hierarchical models. Bayesian Analysis 1: 515–533.
